# The Role of Infant and Early Childhood Gut Virome in Immunity and the Triggering of Autoimmunity—A Narrative Review

**DOI:** 10.3390/diagnostics15040413

**Published:** 2025-02-08

**Authors:** Alexandra Mpakosi, Rozeta Sokou, Martha Theodoraki, Nicoletta Iacovidou, Vasileios Cholevas, Andreas G. Tsantes, Aikaterini I. Liakou, Maria Drogari-Apiranthitou, Christiana Kaliouli-Antonopoulou

**Affiliations:** 1 Department of Microbiology, General Hospital of Nikaia “Agios Panteleimon”, 18454 Piraeus, Greece; 2 Neonatal Intensive Care Unit, General Hospital of Nikaia “Agios Panteleimon”, 18454 Piraeus, Greece; anastasiosmmr@yahoo.gr; 3 Neonatal Department, National and Kapodistrian University of Athens, Aretaieio Hospital, 11528 Athens, Greece; niciac58@gmail.com; 4 School of Medicine, University of Bologna, 40127 Bologna, Italy; billcholevas34@gmail.com; 5 Department of Microbiology, Saint Savvas Oncology Hospital, 11522 Athens, Greece; andreas.tsantes@yahoo.com; 6 1st Department of Dermatology-Venereology, “Andreas Sygros” Hospital, Medical School, National and Kapodistrian University of Athens, 16121 Athens, Greece; a.i.liakou@googlemail.com; 7 Infectious Diseases Research Laboratory, 4th Department of Internal Medicine, Attikon General University Hospital, Medical School, National and Kapodistrian University of Athens, Rimini 1, 12462 Athens, Greece; mdrogari@hotmail.com; 8 Department of Immunology, General Hospital of Nikaia “Agios Panteleimon”, 18454 Piraeus, Greece; c.kalanto@gmail.com

**Keywords:** bacteriophages, gut microbiome, gut virome, autoimmunity, immune system, infant, neonates

## Abstract

**Background**: The bacterial gut microbiome has been the subject of many studies that have provided valuable scientific conclusions. However, many different populations of microorganisms that interact with each other to maintain homeostasis coexist inside the gut. The gut virome, especially, appears to play a key role in this interactive microenvironment. Intestinal viral communities, including bacteriophages, appear to influence health and disease, although their role has not yet been fully elucidated. In addition, bacteriophages or viruses that infect bacteria regulate bacterial growth, thus shaping the composition of the gut microbiome and affecting the immune system. **Infant Gut Virome**: The shaping of the gut microbiome during the first years of life has a significant role in the maturation of the infant’s immune system. In contrast, early dysbiosis has been associated with chronic, including metabolic and autoimmune, disorders later in life. **Purpose**: Although viruses have been shown to be potential triggers of autoimmune diseases, there is a gap in the literature regarding the infant gut virome in autoimmunity development. Despite the lack of evidence, this review attempts to summarize and clarify what is known so far about this timely and important topic in the hope that its findings will contribute to future research.

## 1. Introduction

In the human gut, diverse communities of microorganisms with different functions and properties coexist harmoniously, contributing to the stability of the microbiome. When this balance is disturbed, the gut microbiome is destabilized (dysbiosis), affecting human health. Dysbiosis can dramatically affect the immune response, increase proinflammatory activity, and decrease immune tolerance, leading to autoimmune diseases [1,2,3,4]. Mechanisms involved include abnormal microbial translocation, increased gut permeability, cross-reactivity of microbial components with autoantigens, inflammatory responses, and dysregulated immune cell activation [5].

The gut microbiome includes the bacteriome (bacteria), the virome (viruses), the mycobiome (fungi), the archaeome (archaea), and some parasites that vary during development [6]. In human feces, the predominant virus forms are non-enveloped DNA bacteriophages (approximately 10^15^ bacteriophages) [7]. Tailed phages such as Sipho-, Podo-, and Myoviridae are consistently abundant. Microviridae, single-stranded DNA phages without a tail, are also seen in large numbers [8]. Intestinal bacteriophages are regulators of the bacterial microbiome as they transfer genes, eliminate competing bacteria, simultaneously allow colonization by new prophage-containing bacteria, and may encode toxins that promote bacterial pathogenesis [9,10]. Viruses that grow in human cells are rare in a healthy gut. However, the viral lineages that have been detected include single-stranded DNA viruses such as anelloviruses, cycloviruses, and parvoviruses, and double-stranded DNA viruses such as adenoviruses and papillomaviruses. Of RNA viruses, plant viruses mainly from food sources seem to predominate in a healthy gut [11].

The gut virome has been found to be influenced by environmental stimuli and is, therefore, highly variable between individuals [12]. Even in twin pairs, viromes have been shown to be similar in infancy but to change as they reach adulthood, suggesting the influence of environment and microbiome composition on virome development over time [13]. Another study investigated the viral component of the human microbiome in healthy individuals using metagenomic sequencing of stool samples from four pairs of adult female monozygotic twins and their mothers at three time points over a one-year period. The majority of the virome was shown to be unique to each individual, regardless of familial relationships, and showed high inter-individual variability but intra-individual stability over the time period studied [14].

Alterations and low diversity in the gut virome have been observed in obesity, in type 2 diabetes (T2D), in non-alcoholic fatty liver disease (NAFLD), and in chronic obstructive pulmonary disease [15,16,17,18,19]. In contrast, high gut viral diversity and increases in eukaryotic viruses such as Parvoviridae and Herpesviridae, as well as in *Enterobacteria* phages, *Escherichia* phages, and *Enterococcus* phages, have been observed in patients with alcoholic hepatitis [20]. In addition, several studies have shown that gut virome alterations may trigger the development of several cancers [21,22,23].

Recent studies have shown that the gut virome may also be associated with certain autoimmune diseases. For example, stool analysis of patients with Crohn’s disease and ulcerative colitis has shown an abundance of *Caudovirales*. Similarly, the stools of patients with type 1 diabetes have shown reduced viral diversity, the stools of pregnant women with type 1 diabetes an abundance of picobirnaviruses and tobamoviruses, while those of patients with celiac disease an abundance of enteroviruses [24,25,26,27].

Given that the gut virome plays an important role in shaping bacterial homeostasis, even in early infant life, it has recently been linked to various diseases including necrotizing enterocolitis and asthma [28]. Few studies, however, have focused on the role of infant gut virome in the development of autoimmune diseases. For example, it has been hypothesized that most enteroviruses that cause infections at this age can ascend to the pancreas, where they can create persistent inflammation and islet autoimmunity [29]. Other evidence has suggested that viruses such as enteroviruses, rotaviruses, reoviruses, and influenza virus may trigger celiac disease through molecular mimicry, inflammatory processes, or mediation of interferon (IFN) type 1 [30]. Therefore, the objective of this paper is to fill this gap by reviewing and presenting evidence supporting the association between infant gut virome and the development of autoimmune diseases. Understanding the role of the gut virome in autoimmune diseases is crucial as it offers new insights into the interactions of gut microorganisms that could contribute to the mechanisms of autoimmunity. Furthermore, this review aims to provide an updated overview of the evidence, adding new interesting findings that could be exploited in future research and lead to innovative therapeutic approaches.

## 2. Infant Gut Virome

### 2.1. Infant Gut Virome Composition

Breitbart M et al. were the first to investigate the viral component of the gut microbiome of a healthy one-week-old infant. Metagenomic sequencing of DNA viruses revealed extremely low diversity in the infant gut virome. In the identifiable sequences, mainly phages were found. The authors argued that the most abundant viral sequences did not come from breast milk or formula. They also found that the overall composition of the infant gut virome changed dramatically between 1 and 2 weeks of age, with some sequences remaining stable in the infant’s gut over the first 3 months of life [31]. More recent studies have also agreed that the newborn is born with an intestine free of viruses. Within the first week of life, the neonatal gut has been found to be rapidly colonized, mainly by phages (mostly *Caudovirales* phages) likely derived from induced prophages of gut pioneer bacteria. These early bacteria (mainly *Proteobacteria*, *Actinobacteria*, *Bacteroidetes*, and *Firmicutes*) may come from the maternal gut or vaginal microbiome or from breastfeeding [32,33,34]. In particular, breastfeeding plays an important role in regulating the colonization of the infant intestine with viruses. The feces of infants who start breastfeeding within a few hours of birth have a high virome similarity to the maternal milk they receive, which includes the presence of bacteriophage bifidobacteria, suggesting the possible vertical transmission of the maternal virome to the infant’s gut via breast milk. However, by four months of age, human viruses have been found to be more abundant in stool samples from infants exclusively fed formula when compared to those fed partially or fully on breast milk. Other viruses that have infected the mother can also be transmitted through breastfeeding, including the cytomegalovirus, human immunodeficiency virus, and hepatitis B virus [32].

The initial gut virome is also influenced by other factors such as mode of delivery and antibiotic use. Neonates delivered by vaginal delivery have greater viral diversity than those delivered by caesarean section, with *Caudoviricetes*, *Microviridae*, and *Anelloviridae* dominating their phageome. However, another study recently showed that the diversity of the virome may be lower in neonates born by vaginal delivery than in those born by caesarean section. These results were obtained after sequence analysis and virus whole-genome (WGS) analysis were performed on samples of oral secretions of newborns just seconds after birth, on samples of meconium, and on other body samples from their mothers [35]. In the case of antibiotic use, the diversity of the gut microbiome is reduced, similarly affecting the gut virome due to host loss [36].

Phages increase in abundance rapidly during the first months of life, possibly due to changes in the abundance and diversity of bacterial taxa during this period, development of the infant’s immune system, or acquisition of new phages from the environment [37].

**Figure 1:**
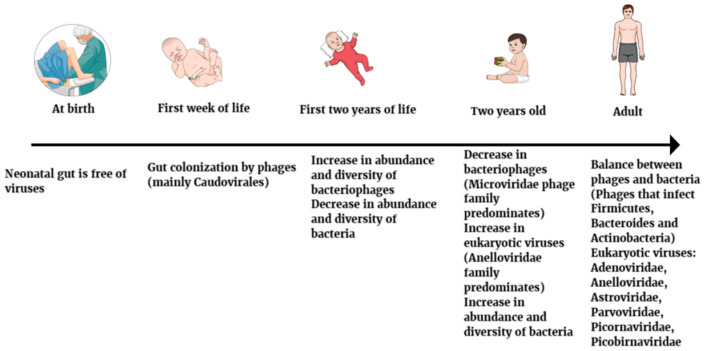
Changes in gut virome composition at different stages of human life.

During the first year of life, continuous shifts occur in the infant gut virome. During the first two years of life, there is an increase in eukaryotic viruses on the one hand and a decrease in the abundance and diversity of bacteriophages on the other. By the age of two, the *Anelloviridae* family of eukaryotic viruses predominates in the viral community. Among the phages, the *Microviridae* phage family predominates [38]. In addition, the reduction in bacteriophages may increase the abundance and diversity of gut bacteria (Figure 1) [39].

Although eukaryotic viruses occupy a minimal part of the healthy gut virome, they may contribute to the pathogenesis of some diseases. Among them are single-stranded DNA (ssDNA) viruses (*Anelloviridae* and *Circoviridae*), double-stranded DNA (dsDNA) viruses (*Adenoviridae*, *Herpesviridae*, *Papillomaviridae*, and *Polyomaviridae*), ssRNA viruses (*Virgaviridae*), and dsRNA viruses (*Reoviridae*). For example, dsDNA viruses (*Adenoviridae*, *Herpesviridae*, *Papillomaviridae*, and *Polyomaviridae*) and some RNA viruses (*Reoviridae*) have been associated with infectious diseases. Moreover, eukaryotic viruses such as echovirus, coronavirus, cytomegalovirus, astrovirus, norovirus, and rotavirus have been associated with the development of neonatal necrotizing enterocolitis (NEC) [36,40].

In addition, despite their small proportion within the gut virome, eukaryotic viruses can influence host immunity. Thus, they can reduce intestinal inflammation by producing interferon-β mediated by Toll-like (TLR) receptor 3 and Toll-like receptor 7. This effect on the immune system has also been suggested in studies with mouse models in which murine norovirus (MNV), which is a common enteric RNA virus, has been found to be able to induce beneficial host responses mediated by the type I interferon response and interleukin (IL)-22 expression, leading to epithelial proliferation and protection against intestinal damage in germ-free or antibiotic-treated mice [41,42].

### 2.2. Factors Affecting Gut Phageome Development and Diversity

As mentioned above, phages are significantly more abundant compared to eukaryotic viruses in a healthy gut virome. Bacteriophages (phages) are prokaryotic viruses that infect bacteria and shape the microbiome through phage–bacteria interaction in the context of co-evolution in different environments. Infection of bacteria can occur through the two cycles of phage replication. On the one hand, the lytic cycle is characterized by infection of a bacterial cell and subsequent bacterial cell death (lysis) leading to the immediate production of new phages. On the other hand, the lysogenic cycle is characterized by the integration of the phage genome into the bacterial genome (prophage), where it remains until induction, lysis, and production of new phages [43].

As also previously mentioned, the neonatal gut is rapidly colonized within the first week of life by phages derived from induced prophages of gut pioneer bacteria, either from the environment, the mother, or breastfeeding [32]. Between birth and 3 years of age, the phage population within the phageome of the infant gut is constantly changing. However, about 9% of the original phages persist during this time period and play a key role in shaping the infant’s gut microbiome. It has been shown that vertically transmitted strains of phage are probably those that remain in both mothers and infants through a continuous and reciprocal seeding between them [44]. Lou YC et al. reported that this persistence of some phages may be due to either co-transmission of their bacterial hosts from the mother, such as *Bacteroides* spp., which are permanent colonizers of the gut, or due to their adaptation to the gut environment or tolerance to the immune system or even due to the evasion mechanisms of bacterial defense systems. However, the lack of *Bacteroides* strains in preterm infants results in fewer long-term persistent phages settling in them compared to full-term infants. Moreover, phages with stop codon reassignment are more likely to persist than non-recoded phages. Lou YC et al. also argued that the phageome of both preterm and full-term infants increases in diversity as the infants grow, and that they reach the diversity of the mothers after 3 years [44]. However, their conclusion contrasts with findings from other studies, which show that gut bacteriophages show a high degree of diversity during the first months of life and gradually decrease over time [45]. Different methodologies and sample selection between studies could explain these differences.

During infancy, the phageome is influenced by various factors, including diet. For example, lactation affects phage populations [32]. In particular breastfeeding enriches the infant gut with beneficial bacteria and associated phages and prevents colonization by eukaryotic viruses [36]. Conversely, fecal eukaryotic viral loads have been found to be higher in formula-fed infants. Nevertheless, a study showed that gut phageome diversity in neonates was significantly lower than in children, possibly due to their exclusive diet of milk or breast milk [46].

Another example is malnutrition which has been shown to greatly disrupt normal phageome. On the contrary, infants with appropriate nutrition create and shape their intestinal microbiome, establish lysogens and free phages, and increase the populations of *Caudovirales* phages, which will later predominate in gut phageome [47]. Indeed, in the intestinal phageome of children, it has been found that phages from the families *Siphoviridae* and *Myoviridae* (belonging to the family *Caudovirales*) and *Microviridae* predominate in greater proportions than in the neonatal phageome [48]. Similarly, phages from the families *Microviridae*, *Siphoviridae*, and *Myoviridae* also dominate adult phageome [48]. However, infants have greater phage diversity and less stability over time in their intestinal phageome compared to that of an adult. Nevertheless, both infants and adults have high interindividual variation in the phageome [49].

Therefore, infancy is a critical period for the development of the phageome. During the first two years of life, phages in the infant gut replicate by the lytic cycle strategy and regulate bacterial diversity and abundance within a dynamic interacting framework. In contrast, phages in the gut of healthy adults replicate by the lysogenic cycle strategy [43]. Although the lysogenic cycle is usually associated with environments of low nutrient content and low bacterial abundance, its prevalence in the gut may mean that phages in this environment can benefit from the high fitness of their bacterial hosts [50]. It has been suggested that in the lumen and upper mucus layer, where the bacterial abundance is higher, the lysogenic cycle predominates, whereas in the inner mucus layer, where the bacterial abundance is lower, the lytic cycle predominates. A mucosal layer disruption could, therefore, trigger further lytic replication, shaping the bacteriome and affecting host immune system [51]. Gut dysbiosis and subsequent inflammation may lead to oxidative stress that may activate the lysogenic–lytic shift [52].

Gut dysbiosis is also closely associated with necrotizing enterocolitis that mainly affects very premature infants. Brunse A et al. showed in a clinically relevant animal model that fecal filtrate, from which they had removed the bacteria from the donor’s feces by micropore filtration and left the bacteriophages, in contrast to fecal microbiota transplantation, could effectively prevent NEC without side effects [53]. In addition, lower helper T-cell and greater naive T-cell responses were observed in filtrate recipients compared with complete microbiota recipients, suggesting that phages may play a role in immune tolerance of gut bacteria in the early neonatal period [53]. Another recent study has suggested that there is a direct association between phages and the host’s immune system, and that they may contribute to immune maturation through TLR-9 [54]. Furthermore, it has recently been hypothesized that bacteriophages could cause autoimmune diseases such as celiac disease by modulating bacterial dysbiosis or by playing an important role in the initiation and exacerbation of intestinal inflammation [55]. However, the role of phages in host immunity is still unclear.

### 2.3. Bacteriophages and Immune System

**Figure 2:**
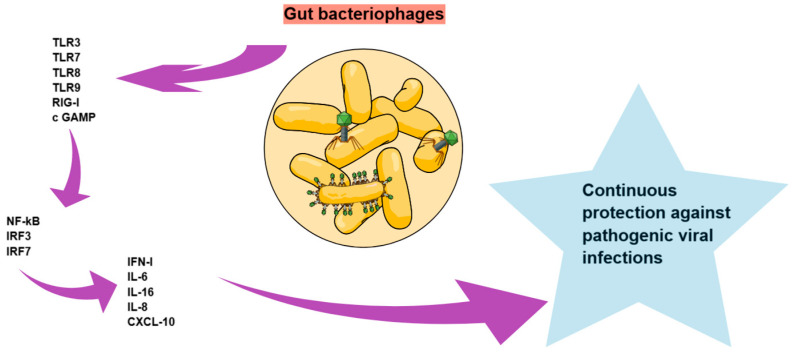
Gut bacteriophages and immune system. TLR: Toll-like receptor, RIG-I: retinoic acid inducible gene I, cGAMP: cyclic-GMP-AMP synthase, NF-κB: nuclear factor-κB, IRF: interferon regulatory factor, IFN-I: type I interferon, IL: interleukin, and CXCL-10: C-X-C chemokine motif ligand 10.

Although the role of bacteriophages in the immune system needs to be further elucidated, evidence has shown that they may induce a humoral immune response. Other studies have also demonstrated that orally administered phages can induce innate and adaptive immune responses in tissues [56]. Within the intestine, cells of the immune system are able to recognize antigenic components or pathogen-associated molecular patterns (PAMPs) such as those produced by viruses, and to subsequently secrete the cytokines responsible for regulating the balance between the virome and immune system [10]. Among the antiviral sensors of the innate immune system, the Toll-like receptors, including TLR3, TLR7, TLR8, and TLR9, occupy a dominant role. In addition, retinoic acid inducible gene I (RIG-I), which is a large protein/pattern recognition receptor (PRR), triggers early innate immune responses against RNA viruses [57]. Moreover, the cycloplasmic DNA sensor cyclic-GMP-AMP synthase (cGAMP) is also involved in virus recognition. Once activated, these receptors then trigger signaling cascades that, in turn, activate the transcription of nuclear factor-κB (NF-κB) and interferon regulatory factors (IRF) 3 and IRF 7, which promote the expression of antiviral molecules including type I interferon, proinflammatory cytokines such as Interleukin-6 and Interleukin-1 beta (b), and chemokines such as Interleukin-8 and CXCL-10 (C-X-C chemokine motif ligand 10) [58]. This cascade of processes can also be triggered by gut bacteriophages, which in this way promote continuous cytokine production that, in turn, leads to stimulation of the antiviral immune response and continued protection against pathogenic viral infections (Figure 2) [9,59]. As mentioned above, bacteriophages are in permanent interplay with gut bacteria. Thus, in certain conditions, such as immunodeficiency, the integration of their genome into bacteria can promote the expression of phage particles, which, in turn, stimulate the immune system. However, bacteriophages possess hypervariable regions in their genome that encode phage tail fiber proteins and immunoglobulin superfamily (IgSF) proteins, giving them the ability to present divergent phage peptide sequences and thereby evade the responses of an adaptive immune system [12].

In addition, some bacteriophages are capable of producing enzymes that modify the O-antigen component of lipopolysaccharide (LPS) of bacteria such as *Salmonella*, *E. coli*, *Shigella*, and *Vibrio cholera*, regulating their antigenicity [56,60].

### 2.4. Eukaryotic Viruses and Immune System

Eukaryotic viruses also play an important role in maintaining gut homeostasis and host immunity. Murine model studies have shown that during intestinal bacterial dysbiosis eukaryotic viruses such as norovirus may possibly contribute to the restoration of both the gut architecture and immunity [61]. In addition, several studies have also reported changes in the gut microbiome following infections by eukaryotic viruses. For example, infants have showed a higher abundance of Fusobacteria and Cyanobacteria at the phylum level and a higher abundance of *Bacillus* spp., *Enterococcus* spp., and *Streptococcus* spp. at the genus level after norovirus infection, compared to healthy infants. Moreover, the microbial diversity has been found to be significantly higher after infection by eukaryotic viruses [62]. Additionally, another study found that infants infected with eukaryotic viruses such as norovirus had significantly higher numbers of Enterobacter cloacae compared to healthy infants [63]. In contrast, another study found that the infant gut microbiome was dominated by Actinobacteria both before and after norovirus infection, with a slight shift toward an increase in the proportion of Bacilli and Clostridia several weeks after the end of the first episode of infection [64]. On the other hand, the gut microbiome contributes to antiviral mechanisms such as interferon (IFN) regulation. Indeed, in mouse model studies, modulating IFN signaling pathways inhibits viral replication in the gut and prevents virus-induced intestinal cell apoptosis and viral spread to peripheral tissues [65]. This stimulation of the immune system through antiviral IFN-γ may lead to regulation of macrophage activation. Studies have also shown that these interactions between viruses and bacteria can induce stress responses in the bacteria and increase the production of bacterial extracellular vesicles [66]. In addition, viral infections can cause changes in the protein and lipid content of the bacteria [67,68].

Within the gut, viruses infect host cells, including epithelial cells, dendritic cells and macrophages within the lamina propria and gut-associated lymphoid tissue (GALT) [69]. The nucleic acid of these viruses is then recognized by these cells via pattern recognition receptors (PRRs), including endosomal TLRs, which signal through MYD88 and TRIF, and RIG-I and melanoma differentiation-associated protein 5 (MDA5), which signal through MAVS, to induce the expression of type I (IFN-I) and type III (IFN-III) interferons [69]. For example, MDA5, TLR7, and TLR3 in myeloid cells are involved in an effective response to norovirus. Similarly, the effective response against rotavirus, which is the main cause of gastroenteritis in children, necessarily involves RIG-I and MDA5 [70]. IFN-I (IFNα and IFNβ) that specifically bind the interferon-α/β receptor (IFNAR1 and IFNAR2 complex) and IFN-III (IFNλ) that binds the interferon lambda receptor (IFNLR1 and IL10R2 complex) stimulate genes that downregulate viral replication and prevent the spread of the virus to neighboring cells [71]. It has also recently been shown that cytosolic Nod-like receptors (NLRs) may play a role in the antiviral response in the gut [72].

**Figure 3:**
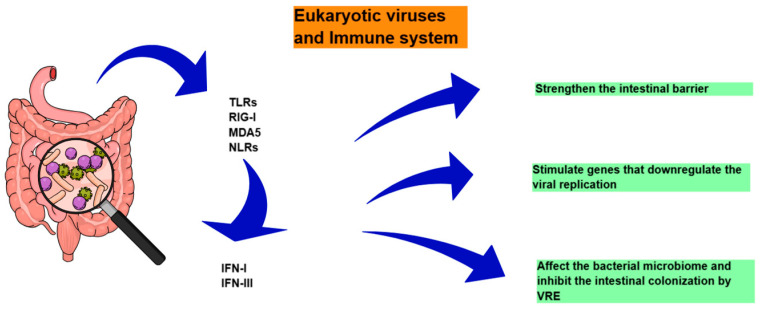
Eukaryotic viruses and immune system. TLRs: Toll-like receptors, RIG-I: retinoic acid inducible gene I, MDA5: melanoma differentiation-associated protein 5, NLRs: Nod-like receptors, IFN: interferon, and VRE: vancomycin-resistant enterococcus.

In any case, the role of viruses in the gut seems to be twofold. On the one hand, intestinal viral infections may contribute beneficially to the immune response, strengthening the intestinal barrier and affecting the bacterial microbiome, as mentioned above. Indeed, in murine model studies, IFN-I signaling following stimulation of the MAVS and RIG-I pathways has enhanced intestinal barrier function [73]. In another study, administration of a TLR7 agonist has stimulated dendritic cells that induce IL-22 production by ILC3s, resulting in the inhibition of intestinal colonization by vancomycin-resistant Enterococcus (VRE) (Figure 3) [74]. In addition, enteric viruses have been detected in healthy individuals, suggesting that enteric viral infections may affect host immunity even when they do not cause diarrheal disease [75,76]. Similarly, murine model studies have shown that norovirus infection can have a beneficial effect on other host systems besides the gastrointestinal tract and can influence immune responses [77,78]. In addition to all of the above, antiviral responses such as IFN-I stimulate beneficial factors for the health of the intestinal epithelium [79].

On the other hand, it has been suggested that excessive production of IFN-I due to viral infection in the gut may enhance the disease. Also, studies have shown that IFN-I treatment for chronic hepatitis C may trigger the development of celiac disease in genetically predisposed individuals who carry HLA-DQ2 or DQ8 alleles [80]. It is also noteworthy that in patients with celiac disease, increased levels of IFN-I production by intestinal DCs are observed, suggesting that viral infections may be involved in the development of this autoimmune disease [81]. In addition, a variant of ATG16L1, a gene involved in cellular autophagy, has been associated with increased susceptibility to Crohn’s disease [82]. Indeed, studies with both Atg16L1 mutant mice and Crohn’s disease patients carrying the ATG16L1 risk allele show morphological defects in Paneth cells. In mutant mice, these defects have been found to be dependent on MNV infection [83]. Crohn’s disease has also been associated with other viruses such as enterovirus as well as a predominance of Caudovirales bacteriophages in the gut [24].

## 3. Early Childhood Gut Virome as Trigger of Autoimmune Diseases

### 3.1. Early Childhood Gut Virome and Type I Diabetes Development

**Figure 4:**
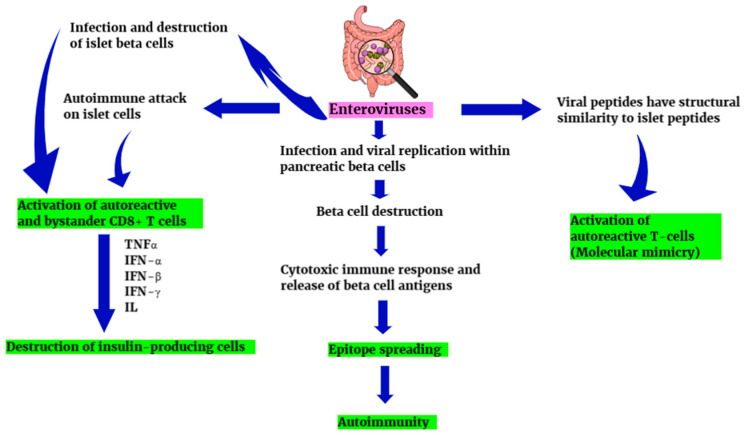
Molecular mechanisms possibly involved in the development of T1DΜ. TNF: tumor necrosis factor, IFN: interferon, and IL: interleukin.

In type 1 diabetes mellitus (T1DM), there is destruction or dysfunction of the functional insulin-producing β-cells of the pancreas in genetically predisposed individuals. Genetic predisposition to T1DM is probably related to several genes. However, among them, the main susceptibility genes for T1DM are identified as HLA complex on chromosome 6 (DR4, DQB*0302 and/or DR3, DQB*0201) [84]. More than 600,000 children under the age of 15 worldwide have T1DM. However, islet autoimmunity (IA) usually precedes T1DM by months to decades. A number of autoantigens have been found to be associated with T1DM, including insulin (IAA), glutamic-acid decarboxylase (GADA), tyrosine phosphatase-like insulinoma antigen 2 (IA2A), islet cell cytoplasmic proteins (ICA), and β-cell-specific zinc transporter 8 (ZnT8A), which can trigger the development of autoantibodies specifically during this period [85,86]. Nevertheless, it seems that genetic predisposition alone is not enough for the development of the disease. Indeed, the most frequently incriminating HLA genotype DR3-DR4 has been found to be associated with only about 40% of T1DM cases. Furthermore, only 30–50% of monozygotic twins appear to be concordant in susceptibility to the disease [87]. On the other hand, more and more studies have suggested that factors other than genetics, such as various drugs, gluten, the gut microbiome, and viral infections may be involved in the development of the disease [88,89,90]. Some of these studies have particularly emphasized the role of viruses in the initiation of the disease [91,92]. For example, IFN-I, particularly IFN-α, and the IFN gene signature have been detected in islets and peripheral blood of T1DM patients. Antiviral responses appear to involve T1DM risk genes in the IFN-I signaling pathway in β cells. It seems that polymorphisms in these genes may cause chronic dysregulated islet IFN signaling, overexpression of IFN-I, the IFN gene signature, major histocompatibility complex class I, and ultimately islet cell inflammation with β-cell apoptosis and potential onset of autoreactivity against β-cell antigens (Figure 4) [92].

**Figure 5:**
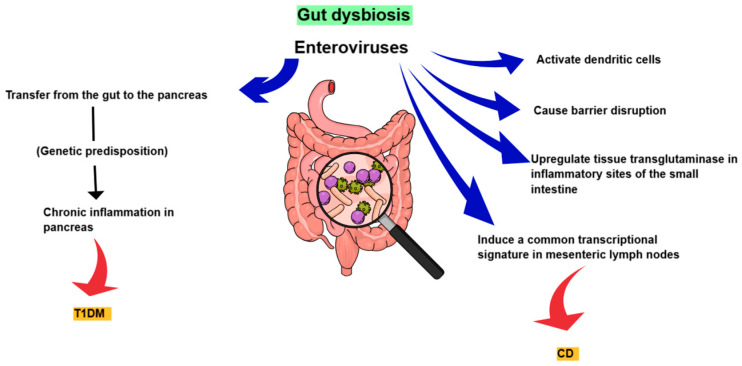
Early childhood gut virome as trigger of autoimmune diseases. T1DM: Type 1 diabetes mellitus and CD: celiac disease.

As mentioned above, gut dysbiosis and the possible transfer of microorganisms from the gastrointestinal tract to the pancreas may trigger the development of T1DΜ (Figure 5). It has also been hypothesized that enteroviruses can potentially be transmitted to pancreatic islets even in young infants. A large study based on a prospective birth cohort analyzed enteroviruses in longitudinal stool samples collected from children with signs of beta-cell destruction and found that enterovirus infections diagnosed by detection of viral RNA in the stools were associated with the development of autoimmunity and islet autoantibody seroconversion, with a time lag of about 9 or more months. In the same study, a predominance of type A enteroviruses, mainly coxsackie A, was observed, a finding that agrees with the results of another birth cohort study [93]. The authors also hypothesized that the small percentage of samples that were positive for coxsackie B, and other B viruses, might be explained by the fact that coxsackie B viruses mainly cause respiratory infections and, therefore, cannot be detected in stool samples. However, other studies have instead supported the role of coxsackievirus B in the pathogenesis of T1D. According to them, the persistence of coxsackievirus B in the pancreas may activate the mechanisms of innate immunity causing chronic inflammation. In this case, and when there is a genetic predisposition, pre-existing autoreactive cytotoxic T lymphocytes can cause insulitis and progressive β-cell destruction. In this context, the gut with persistent coxsackie B virus may be a source of recurrent infections of the pancreas or a cause of immune system damage that could lead to the development of T1DM [94]. In general, however, there are conflicting data from studies from different parts of the world regarding the involvement of enteroviruses in the pathogenesis of type 1 diabetes. Some of them have supported this association, while others have not found a significant correlation [95,96,97,98,99]. However, differences in these findings may be due to differences in methods, study type, study samples, and so on. Nevertheless, a recent systematic review and meta-analysis of observational studies showed a significant association between enterovirus infection and clinical T1DM. According to this review, enterovirus infection may be a more than sixfold risk factor for clinical T1DM. The study also suggests that children younger than 9 years of age have an approximately 34-fold greater risk of developing clinical T1DΜ after an enterovirus infection. In addition, the authors observed that enterovirus infection may be a risk factor for clinical T1DΜ, independent of islet autoantibody positivity [86,100]. Kim KW et al. further argued that viral load may play an important role in disease pathogenesis [101]. Gavin PG et al. combined fecal virome, microbiome, and metaproteome data sampled before and during the onset of islet autoimmunity in 40 children at increased risk of T1DM and showed that microbiota and metaproteome diversity increased with age, while the number and abundance of host antibodies decreased with age. They also showed that there was an interaction between gut microbiome, metaproteome, and virome in young children and that a functional change in the gut microbiome mainly associated with Faecalibacterium contributed to islet autoimmunity [29].

### 3.2. Early Childhood Gut Virome and Celiac Disease Development

**Figure 6:**
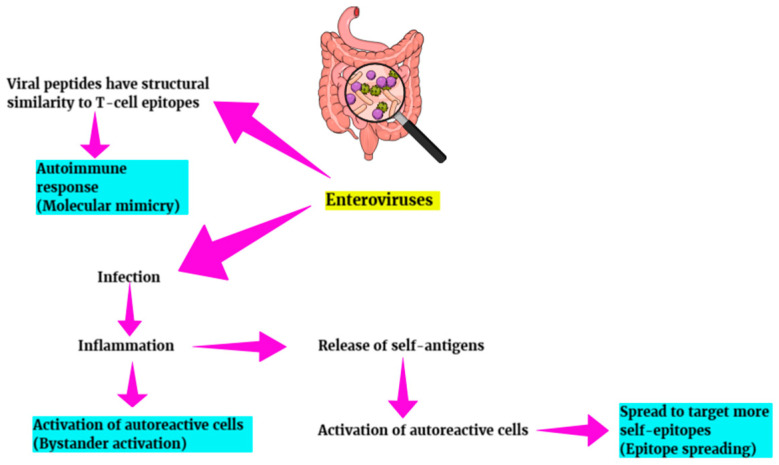
Molecular mechanisms possibly involved in the development of autoimmune diseases.

Celiac disease (CD) is a disease of the immune system associated with the ingestion of gluten by genetically susceptible individuals (those with HLA-DQ2 (DQA1*05:01-DQB1*02:01) or HLA-DQ8 (DQA1*03-DQB1*03: 02) haplotype) and under the influence of unknown environmental factors. Gastrointestinal infections in particular play an important role in the development of celiac disease. During childhood, they can damage the mucosal barrier for transfer of gluten without necessarily causing simultaneous clinical symptoms [102]. Rotavirus, adenovirus, enterovirus, and orthoreovirus have been implicated as potential triggers of CD through different molecular mechanisms (Figure 6) [27,103,104].

Kahrs CR et al. argued that enteroviruses can potentially activate dendritic cells that act as antigen-presenting cells for CD4-positive gluten-reactive T cells in the presence of transglutaminase-modified gluten peptides. They also suggested that patients with celiac disease may have intestinal barrier disruption and sensitivity to enterovirus before developing autoantibodies or that enterovirus may cause barrier disruption, which, in turn, predisposes them to the development of CD [103]. Tapia G et al. also implicated the involvement of parechovirus and the infections it can cause in early childhood in the development of CD in those children who have a genetic predisposition [105]. It has been hypothesized that enterovirus may upregulate tissue transglutaminase in inflammatory sites of the small intestine. It has also been suggested that zonulin, which is a tight junction regulator, is higher in CD patients and correlates with enterovirus density in the small intestinal mucosa of CD cases with severe histological changes [106,107]. Furthermore, Bouziat et al. also showed in mouse models that norovirus and reovirus T1L induce a common transcriptional signature in mesenteric lymph nodes, which is associated with loss of tolerance and CD risk (Figure 5) [108].

Moreover, inflammatory bowel diseases (IBD), such as Crohn’s disease and ulcerative colitis, are chronic inflammatory conditions of the gastrointestinal tract [109]. Research has shown alterations in the virome of individuals with IBD, though no specific viral cause has been confirmed [110]. Some animal-infecting viruses have been proposed as potential triggers, but findings across studies are inconsistent. In pediatric IBD, particularly Crohn’s disease, an increased presence of Caudovirales has been observed, along with a lower abundance of Microviridae, similar to trends seen in adults [24,111,112]. The enrichment of Anelloviridae, common in both pediatric and adult IBD patients, has been linked to the immunosuppressive therapies often used for treatment, suggesting its potential as a biomarker for monitoring immunosuppression [113,114].

## 4. Conclusions

In conclusion, in human feces, the predominant virus forms are non-enveloped DNA bacteriophages. Intestinal bacteriophages play a regulatory role in the bacterial gut microbiome through gene transfer, killing competing bacteria and allowing colonization by new prophage-containing bacteria or by encoding toxins that promote bacterial pathogenesis. Despite their small proportion within the gut virome, eukaryotic viruses can influence host immunity. Enteroviruses can be transmitted to the pancreatic islets even in young infants and may trigger the development of type 1 diabetes (T1D). Gastrointestinal infections with rotavirus, adenovirus, enterovirus, parechovirus, and orthoreovirus in early childhood may also play an important role in the development of celiac disease either by damaging the mucosal barrier for transfer of gluten or by activating dendritic cells that act as antigen-presenting cells for CD4-positive gluten-reactive T cells in the presence of transglutaminase-modified gluten peptides. Virome sequencing has significantly advanced the discovery of the viral candidates linked to pediatric diseases of unknown origin. Current studies provide a broad understanding of how virome changes associate with disease, and future research could clarify causal relationships through controlled animal models. Moreover, large sample cohorts are needed for safer conclusions. Furthermore, the intrinsic characteristics of viruses with their complex life cycles still need to be understood and extensive databases are required to allow mapping of reads to viral genomes. Additionally, analysis of the role of phages will provide insights into the infant gut virome, the microbiome as a whole, and the interactions between them that regulate health and disease. Given the observed negative associations between phage communities and certain diseases, these phages could also be explored as potential therapeutic agents in human trials. This line of investigation holds promise for novel treatment options in the future.

## Data Availability

Data are contained within the article.
